# MDCT and MRI evaluation of cervical spine trauma

**DOI:** 10.1007/s13244-013-0304-2

**Published:** 2013-12-12

**Authors:** Michael Utz, Shadab Khan, Daniel O’Connor, Stephen Meyers

**Affiliations:** Department of Imaging Sciences, University of Rochester Medical Center, 601 Elmwood Avenue, Box 648, Rochester, NY USA

**Keywords:** Cervical spine, Trauma, Fracture, Computed tomography, Magnetic resonance imaging

## Abstract

**Purpose:**

Cervical spine injuries following major trauma result in significant associated morbidity and mortality. Devastating neurological injury, including complete and incomplete tetraplegia, are common sequelae of cervical spine trauma and cause profound and life-altering medical, financial, and social consequences. Most cervical spine injuries follow motor vehicle accidents, falls, and violence. The proliferation of multidetector computed tomography allows for fast and accurate screening for potential bony and vascular injuries. Magnetic resonance imaging is useful for evaluation of the supporting ligaments and the spinal cord after the patient has been stabilised.

**Conclusion:**

Cervical spine injuries are approached with much caution by emergency room clinicians. Thus, it is essential that radiologists be able to differentiate between a stable and unstable injury on MDCT, as this information ultimately helps determine the management of such injuries.

**Teaching Points:**

- *MDCT and MRI are complementary and both may be needed to define injuries and determine management.*
- *MDCT rapidly evaluates the bones, and MRI is superior for detecting ligament and cord injuries.*
- *Injury to one of the three spinal columns may be stable, and injuries to more than one are unstable.*
- *Instability may cause abnormal interspinous and interpedicular distances, or cervical malalignment.*
- *Fractures of the foramen transversarium are associated with vertebral arterial dissection.*

## MDCT and MRI

In most emergency departments, MDCT is the fastest and most practical study for cervical spine injury following trauma. With this technique, a high-spatial-resolution thin-section axial data set can be acquired with reformats in the sagittal and coronal planes in algorithms optimised for evaluation of bones and soft tissues. MDCT is excellent for the timely detection of bony injuries, hematomas involving the paravertebral soft tissues, and signs of subcutaneous soft tissue trauma. MDCT may detect epidural and subdural haematomas; however small collections may be overlooked.

MRI is a critical follow-up study in patients with severe trauma to the cervical spine. MRI is the modality of choice for the assessment of extra-osseous injuries such as epidural haematomas and ligamentous disruption in patients with negative CT studies but a high index of suspicion for injury. Additionally, in patients with confirmed cervical spine injury on MDCT, MRI can more fully evaluate the extent of associated soft tissue injuries. T1 sequences are excellent for surveying the anatomy and caliber of the spinal cord. T2 images with and without fat saturation identify epidural fluid collections, ligamentous disruption, oedema, and herniated discs.

## Anatomy

Injury to the cervical spine is common in major trauma because of the relative lack of supporting structures when compared to the thoracic or lumbar spine. In addition, the wide range of motion of the cervical spine (80-90° flexion, 70° extension, 20-45° lateral flexion, and 90° rotation to each side) and complex kinematics contribute to vulnerability to extreme mechanical forces [1].

**Fig. 1:**
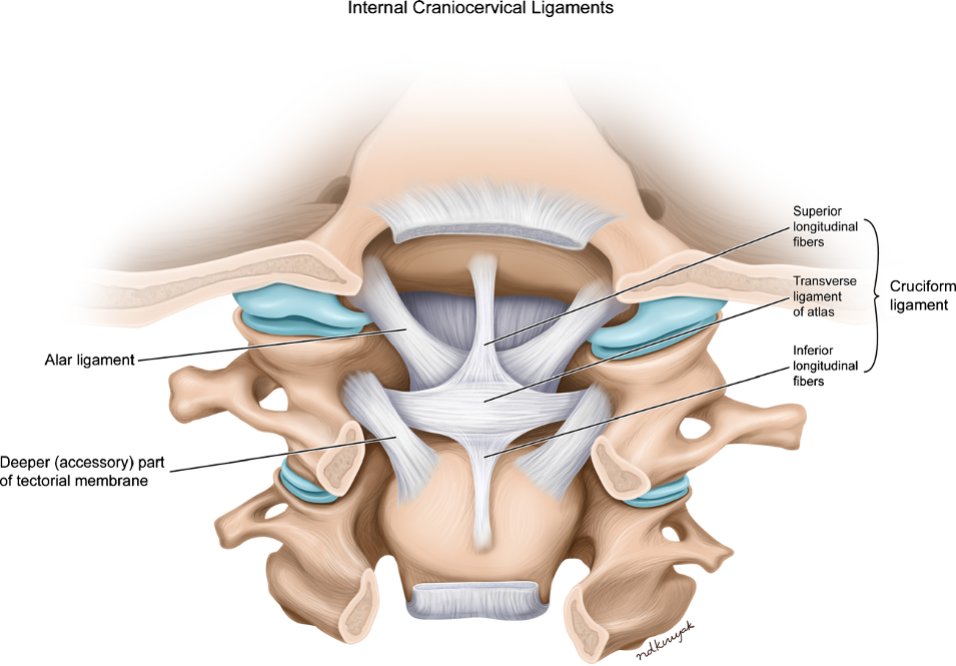
Illustration of a posterior view of the anterior craniovertebral junction demonstrates the important ligaments in this region. The anatomic relationships of the alar ligament and fibers of the cruciform ligament including the transverse ligament of the atlas and superior longitudinal fibers, also called the apical ligament, are depicted

The articulations of the atlanto-occipital (C0-C1) and atlanto-axial (C1-C2) joints are distinct from those of the middle and lower cervical spine. In the atlanto-occipital articulation, the occipital condyles rest within the superior facets of the lateral masses of the atlas. The configuration of the deep articular sockets and tight joint capsule allows for approximately 20° flexion-extension while constraining both rotation and lateral flexion. In the atlanto-axial articulation, the fovea dentis, a small rounded facet in the medial portion of the anterior arch of C1, articulates with the odontoid process of the C2 anteriorly, allowing the atlas and skull to rotate as a unit about the vertical axis of the dens. Three ligaments constrain the movement of the atlanto-axial articulation (Fig. 1). The sturdy alar ligaments extend from the lateral margins of the odontoid process to the medial margins of the foramen magnum bilaterally to limit atlanto-axial rotation. The transverse ligament extends from the medial margins of the lateral masses of the atlas and passes the odontoid process posteriorly in order to secure the odontoid process with the articular facet of C1 anteriorly. The smaller apical ligament extends from the tip of the dens to the anterior margin of the foramen magnum.

The orientation of the uncovertebral and facet joints of the mid and lower cervical spine allows for flexion-extension and rotation at each level while constraining lateral flexion; lateral flexion of the neck is primarily a result of combined rotation and flexion at the C3-C7 levels.

## Fracture stability

**Fig. 2:**
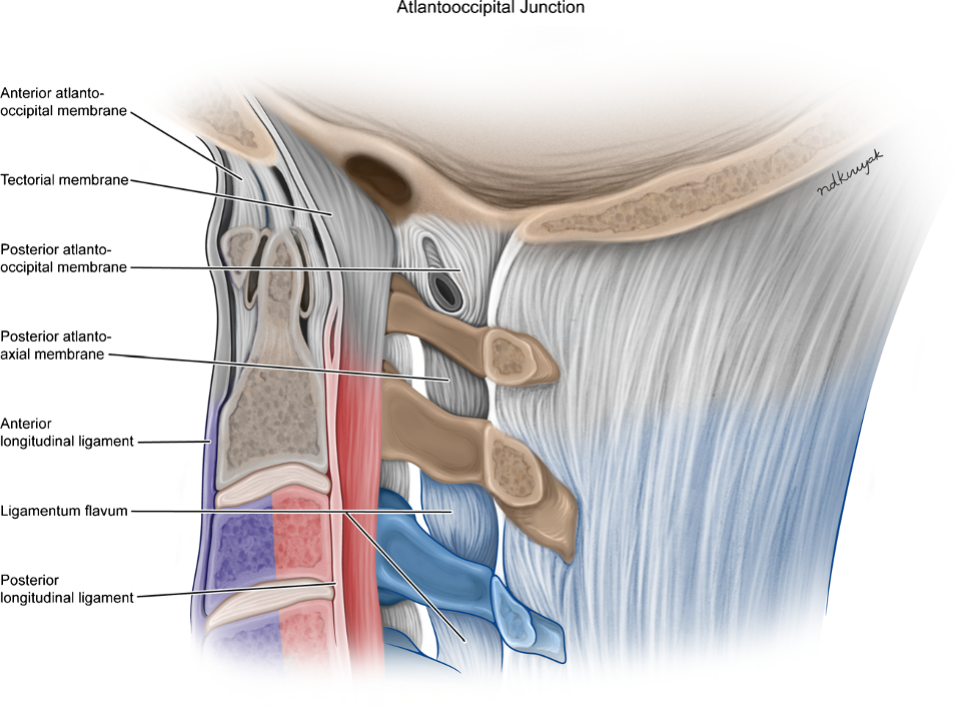
Illustration of a lateral view of the upper cervical spine depicts the three-column model. The anterior column (purple shaded) includes the anterior longitudinal ligament, anterior half of the vertebral body and disc, and anterior annulus fibrosus. The middle column (pink shaded) includes the posterior half of the vertebral body and disc, and the posterior longitudinal ligament. The posterior column (blue shaded) consists of the pedicles, lamina, and spinous processes as well as the ligamentum flavum, interspinous, and supraspinous ligaments

Spinal stability refers to its ability to maintain anatomic alignment under normal stress and loading. Both ligamentous and bony injury can cause loss of stability resulting in further damage to the spinal cord. The most commonly used system for spine stability is the three-column system proposed by Denis [2]. This system divides the spine into the anterior, middle, and posterior columns [3] (Fig. 2). The anterior column consists of the anterior longitudinal ligament, the anterior half of the vertebral body and disc, and the anterior annulus fibrosus. The middle column consists of the posterior half of the vertebral body, posterior disc, and posterior longitudinal ligament. The posterior column consists of the pedicles, lamina, and spinous processes as well as the ligamentum flavum, interspinous and supraspinous ligaments. When two of these columns fail, whether through bony fracture or ligamentous tears, the spine is “unstable”.

**Fig. 3:**
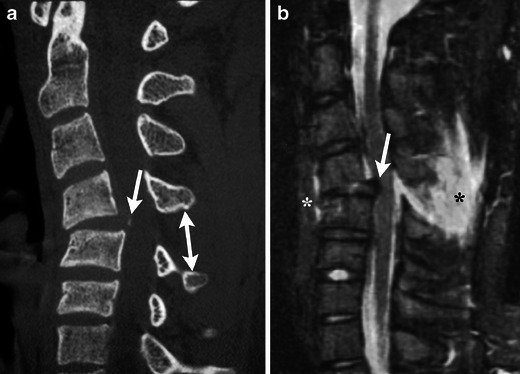
A 16-year-old male who presented with neck pain following a sports injury. (**a**) Sagittal MDCT image demonstrates disruption of the posterior vertebral line, a small osseous fragment with displacement towards the thecal sac (arrow), and increased interspinous distance (double arrow). (**b**) Sagittal STIR image demonstrates disruption of the anterior (white asterisk) and posterior longitudinal ligaments (white arrow) and severe injury to the ligamentum flavum and intraspinous ligaments (black asterisk)

Instability has multiple presentations on CT. There can be widening between the pedicles, facet joints, or spinous processes (Fig. 3). Disruption of the posterior vertebral line, vertebral body subluxation, and loss of vertebral body height greater than 50 % may be indicative of instability. Also kyphosis greater than 20 degrees at a single level may signify an unstable cervical spine injury.

## Upper cervical spine injuries

### Occipital condyle fracture

Occipital condyle fractures occur secondary to multiple mechanisms of trauma. These fractures are commonly classified as described by Anderson and Montesano [4, 5]. Type 1 is a comminuted impaction fracture caused by axial loading and type 2 fractures propagate through the skull base. Both type 1 and 2 injuries are typically stable if unilateral and unstable if bilateral.

**Fig. 4:**
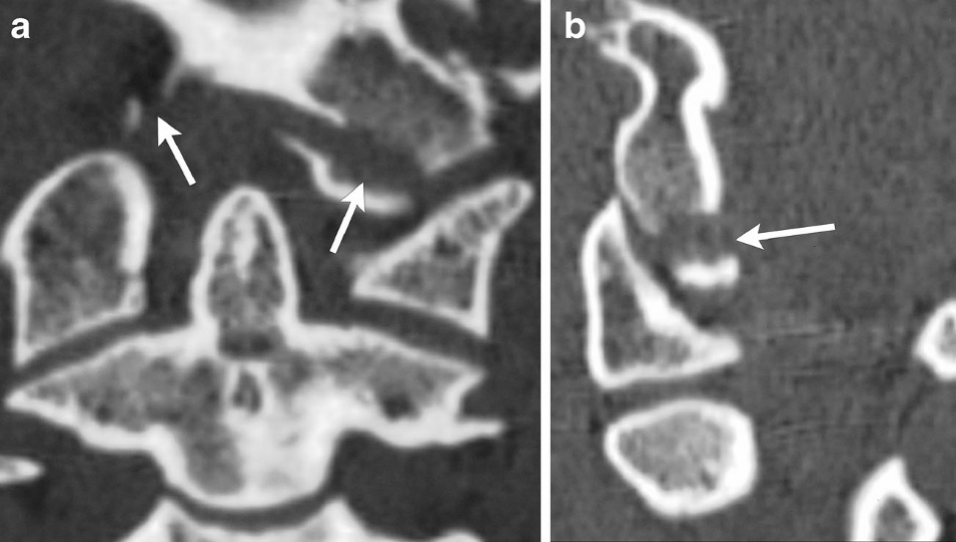
Coronal (**a**) and sagittal (**b**) images of the craniocervical junction after a high-speed motor vehicle collision demonstrate mildly displaced avulsion fractures of the bilateral inferomedial occipital condyles consistent with unstable type 3 occipital condyle fractures

Type 3 is an infero-medial avulsion fracture from tension on the alar ligament (Fig. 4). Type 3 fractures are the most common and often bilateral. Type 3 fractures are associated with instability at the craniovertebral junction due to disruption of the alar ligament and tectorial membrane.

### Atlanto-occipital dislocation

**Fig. 5:**
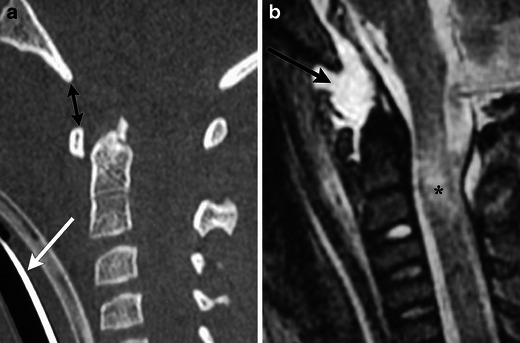
Atlanto-occipital dislocation in a child after a fall from height. (**a**) Sagittal MDCT image in a child demonstrates increased distance between the basion and anterior arch of C1 (black arrows). This patient required intubation (white arrow) because of brainstem and cervical cord injury. (**b**) Sagittal STIR image from another child demonstrates increased T2 signal consistent with alar ligament disruption (black arrow) and upper cervical cord stretch injury (black asterisk)

Similar to occipital condyle fractures, atlanto-occipital dislocations are not unique to any one mechanism of trauma [6, 7]. This injury is more common in paediatric populations because of the relatively higher mass of a child’s head. This unstable injury requires disruption of the ligaments between the occiput and C1, and commonly results in severe stretch or laceration injury to the upper spinal cord and brainstem [8]. Patients with severe injuries usually suffer from neurogenic shock and respiratory arrest; however, with improved management of on-scene injuries and early intubation, survival rates have improved. The key diagnostic feature for this injury is the increased distance between the occipital condyles and C1; this can be identified on coronal and sagittal reformatted images (Fig. 5). MRI may be used to directly evaluate the alar ligaments and tectorial membrane for both tears and capsular oedema.

### Jefferson fracture

**Fig. 6:**
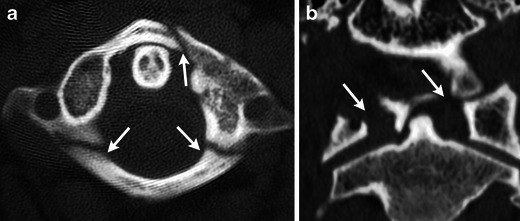
Jefferson fracture. (**a**) Axial CT through C1 after a motor vehicle collision demonstrates a burst fracture involving the left anterior arch and bilateral posterior arches of C1 at their junctions with the lateral masses (white arrows). (**b**) Coronal MDCT image in another patient shows widening of the distance between the dens and the lateral masses of C1 (white arrows)

Severe axial loading injuries can cause burst fractures at the junctions of the anterior and posterior arches of C1 with the lateral masses (Fig. 6) [9]. The combination of anterior and posterior injury is unstable because of its association with transverse and posterior ligament disruption. However, an isolated, unilateral fracture in the anterior or posterior arch may be considered stable. MDCT is excellent for evaluation of bony fragments within the spinal canal.

### Hangman’s fracture

**Fig. 7:**
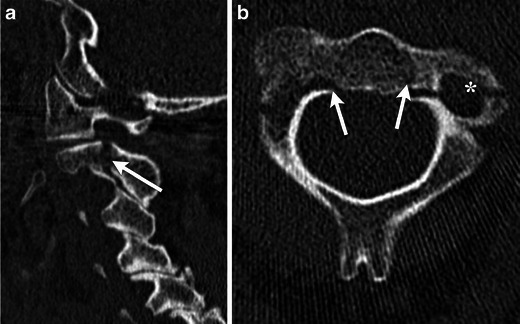
Hangman’s fracture. (**a**) Sagittal and (**b**) axial MDCT images demonstrate bilateral disruption of the pedicles (white arrows) with minimal displacement. Fracture also extends into the left transverse process (white asterisk)

Rapid deceleration and hyperextension injuries can cause fractures of the bilateral pars interarticularis of C2 or traumatic spondylolisthesis of the axis (Fig. 7) [10, 11]. A hangman’s fracture may be stable if minimally displaced (<3 mm), the angle between the fragments is less than 15 degrees, and a normal C2-3 disc space is maintained. Increased displacement or angulation, or widening of the disc space are all indications of injury to the anterior and posterior longitudinal ligament or the C2-C3 disc. Although spinal injury is less common than other levels because of decompression of the canal secondary to pedicle fractures, the presence of unilateral or bilateral facet dislocation is highly associated with neurological complication.

**Fig. 8:**
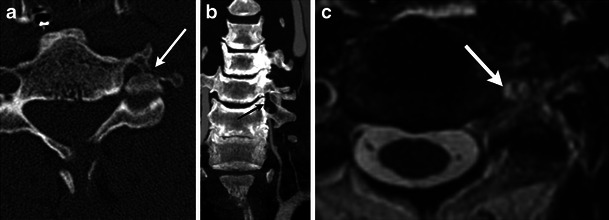
(**a**) Axial MDCT image shows the fracture extending through the foramen transversarium (white arrow). (**b**) Coronal CT angiogram MIP image demonstrates sudden cutoff of the left vertebral artery consistent with dissection (black arrow), which is also seen on the axial T2-weighted image (**c**) as loss of flow void (white arrow)

Fractures of the foramina transversaria are associated with vascular and sympathetic plexus injury. In these patients CT angiography or MR angiography should be considered to evaluate for traumatic dissection of the adjacent vertebral artery [12] (Fig. 8).

### Odontoid fracture

**Fig. 9:**
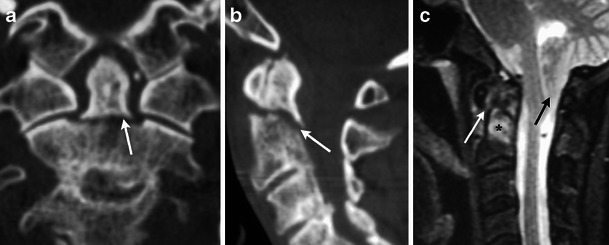
Type II odontoid fracture. (**a**) Coronal and (**b**) sagittal MDCT images demonstrate minimally displaced fracture of the base of the odontoid process (white arrows). (**c**) Sagittal STIR image demonstrates high signal consistent with marrow oedema (black asterisk) and alar ligament injury (white arrow). Incidentally, there is protrusion of peg-shaped cerebellar tonsils below the foramen magnum (black arrow) consistent with a Chiari 1 malformation

**Fig. 10:**
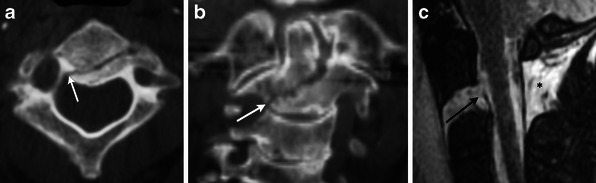
Type III odontoid fracture. (**a**) Axial MDCT image demonstrates a fracture extending from the right base of the odontoid process to the left anterior margin of the body of C2 (white arrows). (**b**) Coronal MDCT image in a different patient shows similar fractures with an oblique path (white arrows). (**c**) Sagittal STIR image in another patient demonstrates severe displacement of the C2 fragments as well as disruption of the posterior longitudinal ligament (black arrow) and posterior ligamentous complex (black asterisk)

Multiple mechanisms of trauma can lead to an odontoid fracture. Typically the fracture is accompanied by posterior displacement of the odontoid fragment and C1 relative to the body of C2. Three types of odontoid fractures exist [12, 13]. Type 1 dens fractures involve avulsion of the tip by the alar ligament and must be distinguished from a well-corticated os odontoideum. Type 2 fractures extend through the base of the odontoid and are associated with a higher incidence of nonunion [14] (Fig. 9). Type 3 fractures are usually obliquely oriented and extend from the base of the odontoid through the body (Fig. 10).

If the fracture fragments are nondisplaced and there is no comminution, all three types of odontoid fractures may be considered stable and initially managed with external cervical immobilisation. Type 2 and type 3 fractures are considered unstable and require surgical fusion if the odontoid fracture is comminuted, the dens is displaced more than 5 mm, or external immobilisation fails to maintain adequate fracture alignment. Due to the higher rate of nonunion, there is a lower threshold for surgical fixation of type 2 fractures, especially in patients over the age of 50.

### C2 body fracture

Fractures that only involve the body of C2 are typically unstable and have a high coincidence with spinal cord injury [15]. A variety of appearances can be seen including sagittal and horizontal plane fractures, burst, and extension teardrop injuries. Management may include nonoperative external fixation in stable, minimally displaced fractures. Surgical fixation with anterior odontoid screw placement and posterior atlantoaxial fusion may be performed if external fixation fails to maintain alignment, there is displacement with ligamentous disruption, or neurologic deficits are present.

### Lower cervical spine injuries

#### Hyperflexion injuries

Hyperflexion injuries can result from falls, diving into shallow water, and dashboard head injuries in unrestrained motor vehicle passengers. Compression of the anterior portion of the vertebral body can cause a range of injuries varying from a simple anterior wedge compression fracture, to a burst fracture, to a teardrop hyperflexion fracture.

**Fig. 11:**
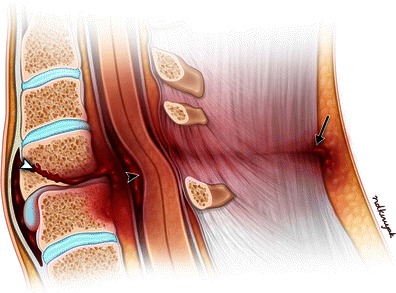
Illustration of a flexion teardrop fracture. In a severe hyperflexion injury compression forces cause an oblique fracture involving the inferior endplate of a vertebral body with disc injury (white arrowhead). There is frequently epidural hematoma formation, which can cause compression of the cord (black arrowhead). The distracting forces experienced by the posterior aspect of the spine cause injury to the posterior ligamentous structures (black arrow)

**Fig. 12:**
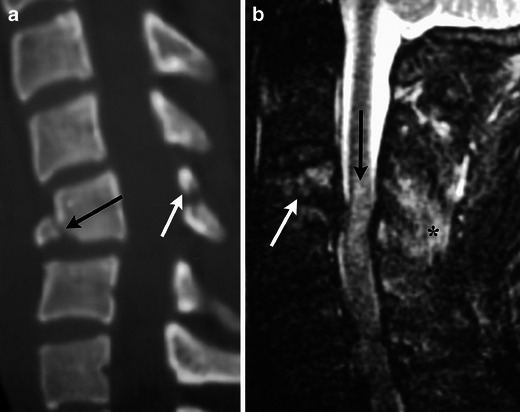
Flexion teardrop fracture. (**a**) Sagittal MDCT image demonstrates fractures of the anteroinferior corner of C5 (black arrow) and horizontally directed fracture through the posterior elements (white arrow), with resultant focal kyphosis and loss of vertebral body height. (**b**) Sagittal STIR image in another patient shows high signal from the fracture (white arrow), within the disrupted interspinous ligaments (black asterisk), and associated cord contusion (black arrow). There is also severe narrowing of the spinal canal

Teardrop hyperflexion fractures are severe injuries with numerous imaging findings (Fig. 11) [16]. The fracture plane extends from the anterior aspect of the inferior endplate and may exit the anterior margin of the vertebral body or superior endplate. The resulting fragment may be triangular or quadrangular in shape. The fractured vertebral body is commonly anteriorly subluxed compared to the inferior vertebral body along with decreased intervertebral disc height (Fig. 12). Both the facet joints and interspinous distances are widened. These unstable injuries are associated with severe ligamentous injury, disc injury, and failure of the facet joints.

**Fig. 13:**
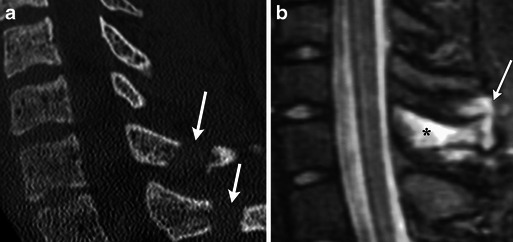
Clay shoveller’s fracture. (**a**) Sagittal MDCT image demonstrates avulsion fractures of C7 and T1 (white arrow). (**b**) Sagittal STIR image demonstrates marrow oedema within the spinous process (black asterisk) as well as injury to the intraspinous and supraspinous ligaments (white arrow)

Less severe flexion injuries, such as lifting a heavy object with the arms extended, can cause shear injury to the supraspinous ligament resulting in an avulsion fracture from the spinous process of C6 and C7, commonly referred to as a clay shoveller’s fracture (Fig. 13) [17]. This stable injury is not confined to C6-C7 and may occur at other levels.

#### Flexion-rotation injuries

**Fig. 14:**
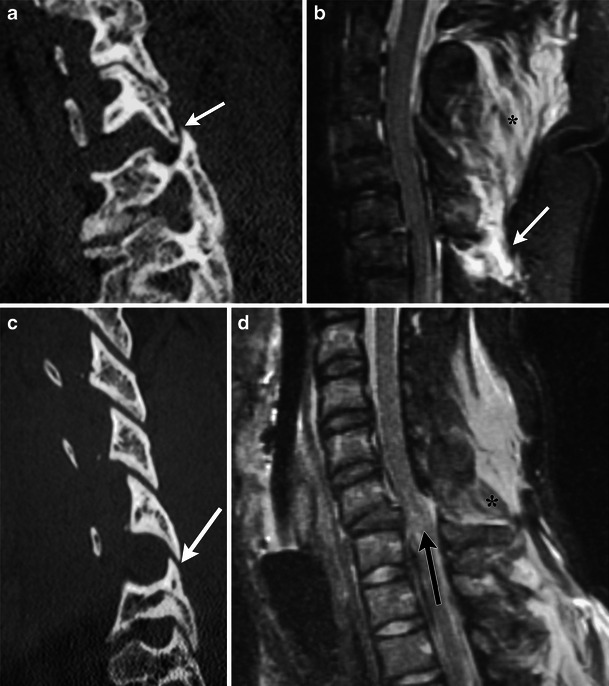
Flexion-rotational injuries: jumped and perched facets. (**a**) Sagittal MDCT image demonstrates anterior “jump” displacement of the inferior articular facet of C6 relative to the superior facet of C7 (white arrow). No associated fracture was present. (**b**) Sagittal STIR image demonstrates increased signal in the ligamentous structures between C6 and C7 (white arrow) as well as diffusely within the soft tissues (black asterisk). (**c**) Sagittal MDCT image demonstrates anterior displacement of C5 with “perching” on the inferior facet of C5 and the superior facet of C6 (white arrow). (**d**) Sagittal STIR image demonstrates cord contusion (black arrow) as well as injury to the posterior ligaments (black asterisk)

Severe hyperflexion with additional rotation causes unstable disruption of the facet-capsular, annular, and longitudinal ligaments with resulting subluxation of the facet joints with or without associated fracture of the facets and vertebral body [18, 19]. On axial CT this appears as rotary subluxation of the facet joint with the “naked facet sign” with or without associated fracture. Sagittal reformats show “jumped” or “perched” facets, which can be unilateral or bilateral (Fig. 14).

#### Hyperextension injuries

Hyperextension injuries can cause fractures of the posterior bony arch including the laminae, facets, and spinous processes [20]. Severe disruption of the anterior longitudinal ligament can occur as a result. Combined anterior ligamentous injury and posterior element fractures result in spinal instability. Associated injuries include cord contusion and traumatic vertebral artery dissection.

## Conclusion

The cervical spine is susceptible to a variety of stable and unstable injuries. Depending on the mechanism of trauma, injuries in this region are associated with high morbidity and mortality. MDCT and MRI are frequently complementary studies in trauma. MDCT is able to identify osseous injuries and assess for cervical malalignment in the acute setting. MRI can further assess injuries in patients with MDCT findings, or assess for occult injury when MDCT is normal. It is critical that the radiologist is familiar with appearances of cervical spine injury on both MDCT and MRI.

## Abbreviations

MDCT: Multidetector computed tomography
MRI: magnetic resonance imaging
